# Short-term sensory and cutaneous vascular responses to therapeutic ultrasound in the forearms of healthy volunteers

**DOI:** 10.1186/2050-5736-2-10

**Published:** 2014-06-02

**Authors:** Shaguftha Sultana Shaik, Joy C MacDermid, Trevor Birmingham, Ruby Grewal, Baseer Farooq

**Affiliations:** 1 Faculty of Health Sciences, Department of Health and Rehabilitation Sciences, Physical Therapy Field, Western University, London, ON N6G 1H1, Canada; 2 Hand and Upper Limb Centre, Clinical Research Laboratory, St. Joseph’s Health Centre, London, ON N6A 4 V2, Canada; 3 School of Rehabilitation Science, McMaster University, Hamilton, ON L8S4L8, Canada; 4 Tier 2 Canada Research Chair in Musculoskeletal Rehabilitation, Faculty of Health Sciences, Department of Health and Rehabilitation Sciences, School of Physical Therapy, Western University, London, ON N6G 1H1, Canada; 5 Hand and Upper Limb Centre, St. Joseph’s Health Care, Division of Orthopedics, Western University, London, ON N6A 4 L6, Canada; 6 Physiotherapist, London, ON N6G 1H1, Canada

**Keywords:** Sensory perception threshold, Skin blood flow, Skin temperature, Ultrasound, Forearm

## Abstract

**Background:**

Therapeutic ultrasound (US) is used for a variety of clinical pathologies and is thought to accelerate tissue repair and help with pain reduction via its thermal and nonthermal effects. The evidence on physiological effects of US on both sensory and vascular functions in humans is incomplete. Hence, the purpose of this study was to determine the short-term impact of two doses of US (3 MHz, 1:4, 0.25 W/cm^2^, 5 min; 1 MHz, continuous, 0.8 W/cm^2^, 3 min), on sensory and vascular responses in the healthy forearms.

**Methods:**

Twenty healthy subjects were recruited (mean age, 29.6 ± 8.8 years) for the study. Superficial blood flow (SBF) in the distal forearms was determined using the tissue viability imaging system. Sensory perception thresholds (SPT) were determined from ring finger (C7, C8) to assess A-beta (at 2,000 Hz) and C fiber function (at 5 Hz), using a Neurometer CPT/C device. Subject’s two hands were randomly allocated to group order (AB/BA). Scores were obtained before and immediately after the application of US and control. Differences in these were analyzed using repeated measures.

**Results:**

Both 3 MHz pulsed US and 1 MHz continuous US showed small to moderate (effect size = 0.12 to 0.68), statistically significant reductions in SBF (3 MHz, mean change = 2.8 AU and 1 MHz, mean change = 3.9 AU, *p* < 0.05 respectively), skin temperature (2.5°C and 1.1°C, *p* < 0.05), and SPT at 5 Hz (1.3 and 1 mA, *p* < 0.05) across time. SPT at 2,000 Hz remained unaltered by all three conditions (*p* > 0.05). Age and gender also had no effect on all outcome measures (*p* > 0.05).

**Conclusion:**

This study demonstrated minor reductions in skin blood flow, skin temperatures, and C fiber perception thresholds immediately after 3 MHz, and 1 MHz US. The responses observed may have been due to a thermo-cooling effect of the gel or due to the direct effect of US on C fibers of median and ulnar nerves. US had a negligible effect on A-beta fibres. This would suggest that future studies looking at physiological effects of US should move towards investigating larger dosages and study the effects in patient populations.

## Background

Therapeutic ultrasound (US) is a physical agent modality that has been used in hand clinics for the management of various musculoskeletal injuries for over 50 years [1-6]. Physiological and therapeutic properties of US are attributed to various nonthermal and thermal responses [4,6-9]. The tissue response to nonthermal ultrasound includes acceleration of tissue healing through cavitation and its associated effects [3,6,8,10,11], while the responses to thermal ultrasound include increases in tissue temperature at superficial and deep levels such as tendons, ligaments, joint capsules, and fascia without overheating underlying fat [3,7,10-13]. Both continuous and pulsed ultrasound are thought to show nonthermal effects and accelerate tissue repair [1-3,6,11,14,15], while continuous ultrasound is thought to add additional therapeutic effects due to heating [6,11,13,16]. Although US has been used for decades, the lack of definitive studies defining its benefit in different musculoskeletal conditions [4,8-10,17,18] has questioned the traditional view of its therapeutic benefits [10]. Multiple systematic reviews and meta-analyses of US has failed to provide definitive conclusions about the effectiveness (or lack of effectiveness) of US because of insufficient evidence [4,6,8-10,17,18]. Several reviews also report disagreement and confusion about the most efficacious treatment parameters for US [4,6,8-10,17,18,20]. Despite this lack of strong evidence, previous reports on US usage in the form of questionnaires and surveys have shown that US is being used frequently in the physiotherapy clinical practice for musculoskeletal conditions [9,21-23,25]. It was found that over 70% to 95% experienced and advanced practice clinicians continue to use US regularly for specific impairments encountered in orthopedic and sports settings, indicating that US is perceived as an important component in the management of selected impairments [9,21-25]. Although dosage is based on the theoretical rationale, there are insufficient dosage trials to define the optimal dosage of ultrasound across different conditions [4,6,9,19,21].

Ultrasound is thought to affect tissue thermodynamics and as such might result in changes in the peripheral circulatory system. A therapeutic modality capable of altering peripheral circulation could affect the health of human tissue and facilitate tissue healing [8,20]. The forearm skin (nonglabrous) is innervated by sensory nerves and sympathetic vasoconstrictor and vasodilator nerves, which respond to thermal, chemical, and mechanical stimuli to provide feedback to the central nervous system and influence cutaneous arteriolar tone (vasoconstriction or vasodilation) via the release of neuropeptides and other vasoactive agents [26-28]. After injury, symptoms of pain can occur because of vascular insufficiency which affects metabolic function in the injured soft tissue or edema and muscular strain, etc. [29]. Alterations in pain can also be related to neural transmission in sensory and pain fibers [29]. Better understanding of the biological effects of ultrasound should include monitoring all these pathways (vascular and neural).

There is currently a scarcity of published clinical trials that have looked at both the neural and vascular responses to ultrasound therapy. Noble et al. used laser Doppler flowmetry (LDF) to assess cutaneous blood flow and concomitant measures of ambient and skin temperatures after applying 3 MHz pulsed (1:2) and 3 MHz continuous US at an intensity of 1 W/cm^2^ for 6 min over the mid-forearm [30]. The authors noted that after sonation, there was an increase in skin blood perfusion with pulsed US and continuous application of US without any significant difference in skin temperature between the US groups. However, these authors measured skin blood flow distal to the US application from a single vessel (single point LDF) instead of the tissues directly affected by the treatment. Further, they did not include sensory monitoring to establish changes in nerve function with ultrasound application [30].

The effects of US are thought to vary with the type of tissue, site, and the dosage used [6,8,11,12,19-21,31]. There is a scarcity of information regarding the effects of ultrasound on hemodynamics resulting from altered treatment times, intensities, and frequencies in human subjects. These evaluations might inform our understanding for potential mechanisms of therapeutic effect that operate through beneficial effect on skin blood flow and sensory function. Hence, the purpose of this study was to determine the effects of two different doses of US (3 MHz pulsed and 1 MHz continuous) on superficial blood flow, skin temperature, and sensory perception thresholds in the distal forearms of healthy volunteers. A secondary purpose was to determine if the responses were affected by age and gender.

## Methods

### Participants

The sample size required for this research was based on the number needed to detect a moderate effect size according to Cohen [32]. A moderate effect (ES *r* = 0.50) using two-tailed alpha (*α* = 0.05) at 80% power requires a sample of 28 participants in each group for a between-subject design and a sample of 14 participants for a within-subject design. As this is a within-subject design and variance within individuals is less than between subjects, a sample size of 20 was considered and approved by the Ethics Board. Statistical significance was considered if *p* < 0.05.

Subjects were recruited by poster advertisement and word of mouth in the university campus. Testing was done in Hand and Upper Limb Research Lab, at St Joseph’s Health Care, London, Ontario, Canada. Healthy subjects aged 18 to 50 years with no recent injury or disease at neck, shoulder, elbow, wrist, or hand within the past year were included in the study. Subjects were divided into two age categories: 18–34 and 35–50 years. There were ten males and ten females in total. Please refer to Table 1 for subject demographics. All subjects were informed to refrain from exercise and drinking beverages 4 h prior to testing. Subjects were excluded if they had ecchymosis, skin infection, open wound, swelling, neurovascular injuries, deficits in sensation in the area to be treated (sensory test to identify sharp and dull sensation; hot or cold), decreased circulation (digital patency test for fingers), pregnancy, presence of a pacemaker/monitoring device, malignancy, hypertension, and cardiac failure. This study was approved by the Western University Research Ethics Board. All participants read the letter of information, had his/her questions answered, and signed a consent form prior to participation in this study.

**Table 1:** Demographic characteristics of participants

**Age, years (M ± SD)**	**29.6 ± 8.83**
**Gender**	
Females, *n* (%)	10 (50%)
Males, *n* (%)	10 (50%)
**Dominance**	
Right, *n* (%)	19 (95%)
Left, *n* (%)	3 (5%)

*M* mean, *SD* standard deviation, *n* number of participants.

### Equipment

#### Ultrasound machine

A ‘Phyaction U’ ultrasound machine (GymnaUniplay N V, Pasweg 6A, and BILZEN) with the capabilities for 1 and 3 MHz frequency operation was used to deliver the ultrasound treatments. The transducer, model U92, had an effective radiating area of 4.0 cm^2^ and a beam nonconformity ratio (BNR) of <4.0 and was calibrated before research was initiated. An aqueous Eco Gel (Eco-med Pharmaceuticals, Mississauga, ON, Canada) was used as the coupling medium for all treatments.

We could not find any clear standard guidelines for US dosage in the literature [21,31]; hence, we adopted the framework proposed by Tim Watson [20,33]. Based on the previous evidence on the effectiveness of ultrasound, he put forth a framework for the treatment parameter selection [33]. The basic principle is that the more acute and irritable the tissue in question, the lower the required dose to achieve a stimulating effect. The frequency selection (1 or 3 MHz) will influence the effective treatment depth (3 MHz is more superficial, i.e., a depth of approximately 2 cm, 1 MHz is effective to a depth of 4 or 5 cm). The pulse ratio needs to be higher for the more acute lesions (1:4) and lower for the more chronic (1:1 or continuous). Intensities vary from 0.1 to 0.3 W/cm^2^ for the acute lesions to 0.4 to 0.7 or 0.8 W/cm^2^ for the chronic lesions. Treatment times are based on the principle of 1 min of ultrasound per treatment head area [20,33]. Hence, we derived two experimental dosages from the above framework which may be applicable for tissue healing in an acute condition (3 MHz, pulsed US, for a lesion <1 cm depth) and a chronic condition (1 MHz, continuous, for a lesion <3 cm depth) at the distal forearm. Subjective skin warmth was used as an indicator of tissue heating and dose selection [6,34].

#### TiVi 600 polarization spectroscopy camera

The tissue viability imager (TiVi, version 7.4 Wheels Bridge AB, Linköping, Sweden) is a small and portable device for high-resolution instantaneous imaging of red blood cell (RBC) concentration in upper human dermal tissue. TiVi software was used to quantify RBC concentration on the anterior aspect of the distal forearm on both arms using a digital camera (Canon Rebel EOS model 450D, Tokyo, Japan) with a polarization lens. The camera was adjusted to point downwards and parallel to the surface of the desk. A royal blue-colored cushion was used to rest the forearm and to fill the camera view. An outline was drawn to standardize hand positioning. The participants were positioned with their shoulder in neutral, elbow in 90° flexion, wrist in neutral position, and the forearm fully supinated and placed approximately at the level of the heart. Each image was captured with the polarized lens set at the ‘cross polarization’ setting and the camera was positioned at a distance between 30 cm from the participant’s hand. Image quality was set to ‘medium normal’. The camera has a light penetration depth between 0.4 to 0.5 mm [35], and this light contains information about the main chromophores in the epidermis (melanin) and dermis (hemoglobin), while the surface reflections contain information about the surface topography, such as texture and wrinkles. Once the images were captured, they were processed using the TiVi software.

For each participant, one image at baseline and immediately after US therapy and control (rest) were used for processing and analysis. There were a total of eight images per participant (four in each arm). Regions of interest (ROI) were selected over the treatment area (2× effective radiating area or ERA) at the distal forearms. The magnitude of RBC concentration over the selected ROI’s was obtained using ‘image analysis’. Values for the TiVi are measured by arbitrary units (AU) as defined by the manufacturer. Data was first exported from the TiVi software into Microsoft Excel and then imported into SPSS version 20.0 for statistical analysis. The technique has shown many uses in drug development, burn investigations, pressure studies, and general research maneuvers due to the ease of use, portability, and low cost [35,36]. The TiVi has been validated for construct validity to measure superficial RBC concentrations with *in vitro* fluid models and computer simulations. TiVi software is able to accurately calculate the oxygen saturation level of 91.5% *in vivo*, which is within the physiological range of oxygen saturation within blood [35,37]. It has been shown to be sensitive to change during blood occlusion testing [35], and drug testing on skin [38], and has also demonstrated good inter-laboratory reliability [39].

#### Neurometer® CPT/C device

The Neurometer® CPT/C device (Neurotron Inc., Baltimore, MA, USA) evaluates sensory nerve conduction from the periphery to the brain and has been shown to detect differences in neural function in asymptomatic subjects when neural stress was administered [40]. This portable battery-operated nerve stimulator has the ability to emit three different frequencies in sinusoidal waveforms to selectively target different subpopulations of nerve fibers dependent upon nerve fiber diameter. A frequency of 2,000 Hz is used to stimulate the large myelinated A-beta fibers (A_β_ fibers) which detect cutaneous touch and pressure; a 250-Hz stimulus will stimulate myelinated A-delta nerve fibers (A_δ_ fibers) which are mechanoreceptive and detect fast pain, pressure, and temperature, while a frequency of 5 Hz is used to stimulate the small unmyelinated C-polymodal nociceptive fibers which detect slow pain and temperature and are postganglionic sympathetic fibers. This device has been used in numerous studies to detect, screen, and diagnose the abnormalities of peripheral nervous system and normal ranges for all nerve fiber types (A-beta, A-delta and C fibres) that have been established to assess normal sensation, increase in sensation (hyperesthesia), decrease in sensation (hypoesthesia), and no sensation at all (anesthesia) [41,42]. The neurometer has been shown to be both specific (73%) and sensitive (74%) in the clinical examination of carpal tunnel syndrome and is considered a reliable and valid measure of quantitative sensory function [43].

Ranged CPT (R-CPT) is a sensory perception threshold test which can be completed in 3 to 6 min for each test site. It is typically used to confirm or rule out sensory involvement in large samples such as in screening and monitoring therapeutic outcomes [41,42]. In R-CPT, each frequency is repeated several times to ensure accuracy and reproducibility. The average time needed to complete the tests is reported to be less than 10 min [41,42]. The neurometer reports values as the normal range (R-CPT level, 6–13), hyperesthesia (R-CPT level, 1–5), and hypoesthesia (R-CPT level, 14–25) [41,42]. Sensory nerve perception thresholds at two frequencies, 2,000 and 5 Hz, to test two different nerve fibers that were used in this study. To begin 2,000-Hz stimulation, the skin was cleaned with a skin paste and then the 1 cm gold electrodes coated with small amount of gel were attached to the ring finger (area innervated by C7,C8) with an adhesive tape. Then, the participants were asked to press and hold the red ‘Test cycle’ button on the remote control box and release it as soon as they begin to feel the tingling or buzzing sensation. The machine records the response when the button is released and the same process is repeated 7–10 times until a score is displayed. In total, three scores are obtained at 2,000 Hz. The same procedure is repeated at 5 Hz. These test cycles end automatically after few repetitions (7–10 times), and the machine displays score for 5 Hz.

### Experimental procedure

#### Randomization

**Figure 1:**
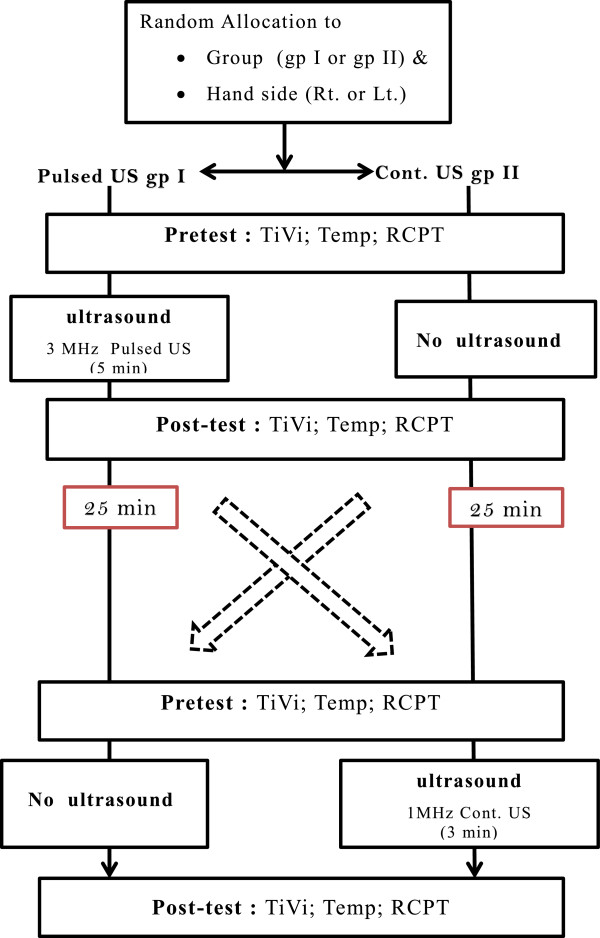
Flow chart for the study design (cross-over AB/BA).

We used a randomized cross over, repeated measures design in this study. Random allocation to treatment order and hand was achieved through subject’s selection of assignment contained in an opaque envelope. After explaining the protocol and obtaining consent, each participant was asked to pick up two different colored sealed envelopes from two bundles, which had information on the side of hand and sequence of therapy to be initiated. Once the hand side was picked, the participant was then randomized to either pulsed US group or continuous US group based on the labeling on cards inside these envelopes. There were three conditions for testing: a control condition (or rest), pulsed ultrasound, and continuous ultrasound. If the card showed pulsed US group, then the order of therapy was pulsed US followed by rest in the hand first selected. After this, the opposite hand received therapy in reverse order (continuous US group), beginning with rest and then followed by the continuous US. A 25-min gap [13] was established between each treatment condition (control/rest, pulsed, and continuous US) to provide a washout period and to minimize any potential carry over effects of US. Each hand acted as its own control. Therapy instructions and outcome assessments were all provided by a single physiotherapist. The two group sequences were completed on the two hands one after the other on the same day. The flow chart outlining the study design is in Figure 1.

#### Ultrasound protocol

After acclimatization to room temperature for 10 min, participants were first measured using the TiVi over the treatment area in distal forearms. This was followed by skin temperature measurement using King’s infrared digital thermometer. Then, a range-CPT test was recorded from the tips of ring finger (over C7/C8 dermatome level) to assess sensory perception thresholds at 2,000 Hz (for A-beta fibres) and 5 Hz (for C fibres). These three measurements (TiVi, temperature, R-CPT) were done before (pretest or baseline) and immediately (post-test) after each control condition/rest and ultrasound application in similar order. After completing baseline assessments, based on the group order selection, participants either underwent therapy with continuous US or pulsed US or were informed to rest for 3 to 5 min without any treatment, during the control/rest conditions. The next therapy was initiated after 25 min during which the skin temperature returned to its pre-treatment level. To ensure that the ultrasound was directed at the target tissue, a template equivalent to twice the area of ultrasound applicator (2× ERA) was placed on the anterior aspect of distal forearm (distal border coinciding with the ulnar styloid process). Pulsed US was delivered at 3 MHz pulsed mode, with 1:4 duty cycle, at an intensity of 0.25 W/cm^2^ for 5 min. Whereas, continuous US was delivered at 1 MHz, continuous mode, with an intensity of 0.8 W/cm^2^ for 3 min (adapted from T. Watson) [15,33]. The ultrasound applicator was moved back and forth (circular motion) in the template at a rate of 3 to 4 cm/s continuously and was timed with a metronome. Instructions were given to the participants to inform if the therapy was uncomfortable, in order to stop the machine and note the time of discomfort-free therapy. After US therapy, the treatment area was cleaned with a towel and then TiVi, skin temperature, and R-CPT responses were measured immediately after (post-test). The same process was repeated after control/rest.

TiVi software was used to calculate the mean blood flow (AU) in the treatment area and then the data was transferred to an Excel sheet (Microsoft 2010). The sensory perception thresholds obtained at 2,000 and 5 Hz (mA) were recorded directly from the digital display of the Neurometer CPT/C device along with temperature (°C) readings onto a separate data collection sheet. Subjects were instructed to keep the area clean and covered and to self-monitor for any signs of local skin irritation after they leave.

### Data analysis

Data was entered in SPSS and random checks of 10% of the data against the hard copies of data sheets was used to ensure data quality (100% were correct so no further data audits were performed). Descriptive statistics were run to further investigate data quality and assess data normality. The majority of data was normally distributed as assessed by Kolmogorov-Smirnov test (*p* > 0.05); hence, parametric tests were used for quantitative data analysis. The outcome measures (RBC concentration, temperature, and sensory perception thresholds) were assessed for differences using general linear models, repeated measures (GLM, RM using SPSS version 20, IBM Inc., Chicago, IL, USA). Models assessed whether there were differences between baseline and immediately after control or ultrasound therapy (1 and 3 MHz). Interactions were examined for significance between time and treatment group. *Post hoc* analyses were performed using Bonferroni correction wherever necessary. Pairwise comparisons were used to perform within-group comparisons for treatment and control. The GLM model was run without covariates and then repeated with age and gender as a covariate to test for differential responses. Significance level was set at *p* < 0.05 level unless otherwise noted. All values were expressed as means, standard deviation, and confidence intervals.

## Results

Twenty healthy volunteers were recruited between November 2012 and February 2013. No data points were missing. Demographic information of participants is presented in Table 1. The group means, standard deviation (SD), 95% confidence interval (CI), effect size (ES), and change scores (CS) for skin blood flow, skin temperature, and sensory perception thresholds at 2,000 and 5 Hz are shown in Tables 2 and 3 and are summarized by outcome measure in the following.

**Table 2:** Summary of results for skin blood flow and skin temperature over the treatment area

	**Pulsed US group**												**Continuous US group**											
	**Pulsed US (3 MHz)**^**a**^						**Control-p**						**Control-c**						**Continuous US (1 MHz)**^**b**^					
	**Before**		**After**		**CS**	**ES**	**Before**		**After**		**CS**	**ES**	**Before**		**After**		**CS**	**ES**	**Before**		**After**		**CS**	**ES**
Bf	M	60	M	57.6	2.8*	0.12	M	60.3	M	60	1.1*	−0.07	M	60.6	M	59.9	0.8*	0.03	M	60.6	M	56.9	3.5*	0.2
	SD	10.6	SD	11.2			SD	10.7	SD	8.8			SD	8.9	SD	9.9			SD	8.9	SD	10.6		
	CI	(55–65)	CI	(52–62)			CI	(55–64)	CI	(57–66)			CI	(56–64)	CI	(54–64)			CI	(56–64)	CI	(51–61)		
St	M	33.1	M	30.9	2.1*	0.68	M	33.1	M	33	0.1*	−0.04	M	33.4	M	33.1	0.3*	0.2	M	33.1	M	32.1	1.0*	0.42
	SD	1.1	SD	1.1			SD	1.2	SD	0.8			SD	1.0	SD	0.9			SD	1	SD	1.2		
	CI	(33–34)	CI	(30–32)			CI	(33–34)	CI	(33–33)			CI	(33–34)	CI	(33–34)			CI	(32–34)	CI	(31–33)		

^a^Pulsed mode, 3 MHz, 2.5 W/cm^2^, 5 min.; ^b^continuous mode, 1 MHz, 0.8 W/cm^2^, 3 min. *Control-p* no US therapy in pulsed US group, *Control-c* no US therapy in continuous US group, *Tx* treatment, *M* mean, *CI* 95% confidence interval, *SD* standard deviation, *CS* change scores, *ES* effect size *r* (pre-post/pooled SD), *Bf* skin blood flow, *St* skin temperature. *Significance level at *p* < 0.05.

**Table 3:** Summary of results for sensory perception thresholds at 2,000 and 5 Hz from ring finger

	**Pulsed US group**												**Continuous US group**											
	**Pulsed US (3 MHz)**^**a**^						**Control-p**						**Control-c**						**Continuous US (1 MHz)**^**b**^					
	**Before**		**Aftre**		**CS**	**ES**	**Before**		**After**		**CS**	**ES**	**Before**		**After**		**CS**	**ES**	**Before**		**After**		**CS**	**ES**
Aβ	M	8.5	M	7.6	0.9	0.14	M	8.5	M	7.1	1.3	0.12	M	8.6	M	7.8	0.8	0.2	M	8.6	M	8.0	0.7	0.1
	SD	3.8	SD	1.7			SD	3.8	SD	1.8			SD	2.4	SD	1.7			SD	2.4	SD	2.2		
	CI	(6.8–10)	CI	(7.5–9.7)			CI	(6–10)	CI	(7–8)			CI	(6–8)	CI	(7–9)			CI	(7–9)	CI	(6.9–9)		
C	M	13.4	M	12.2	1.3*	0.17	M	13.4	M	10.5	2.8*	0.36	M	12	M	10.8	1.6*	0.2	M	12.4	M	11.4	1*	0.1
	SD	3	SD	3.6			SD	3	SD	3.8			SD	4.3	SD	3.9			SD	4.4	SD	3.6		
	CI	(12–15)	CI	(10–13)			CI	(12–14)	CI	(8–12)			CI	(10–15)	CI	(9–13)			CI	(10–14)	CI	(9.8–13)		

^a^Pulsed mode, 3 MHz, 2.5 W/cm^2^, 5 min.; ^b^continuous mode, 1 MHz, 0.8 W/cm^2^, 3 min. *Control-p* no US therapy in pulsed US group; *Control-c* no US therapy in continuous US group, *Tx* treatment, *M* mean, *CI* 95% confidence interval, *SD* standard deviation, *CS* change scores, *ES* effect size *r* (pre-post/pooled SD). Aβ = R-CPT at 2,000 Hz; C = R-CPT at 5 Hz; *R-CPT* ranged current perception threshold test. *Significance level at *p* < 0.05.

### Sensory perception threshold at 2,000 Hz from ring finger

**Figure 2:**
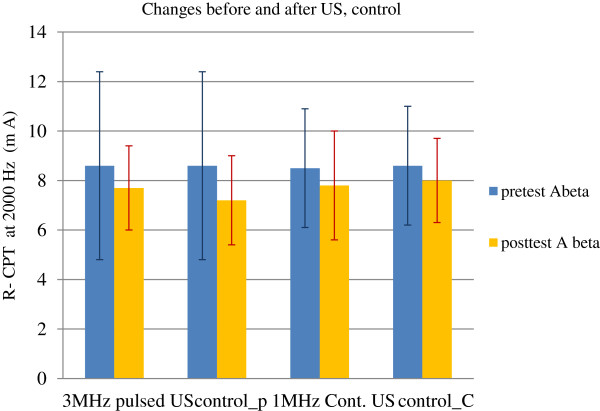
Sensory perception thresholds at 2,000 Hz before and after US and control.

No significant difference in sensory perception threshold at 2,000 Hz was observed in the ultrasound or control, across the time points. There was no significant interaction between time and treatment (Table 3 and Figure 2).

### Sensory perception threshold at 5 Hz from ring finger

**Figure 3:**
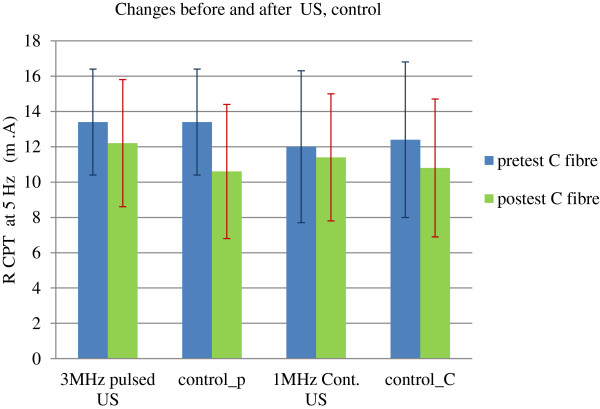
Sensory perception thresholds at 5 Hz before and after US and control.

A significant decrease in perception threshold at 5 Hz was observed from pretest to post-test with US (either 1 or 3 MHz) as well as control condition (*p* < 0.05). A significant interaction was also found between time and treatments (*p* < 0.05). *Post hoc* comparisons revealed that control condition resulted in a statistically significant decrease in threshold (13.4 to 10.6 mA) when compared to pulsed US at 3 MHz (13.4 to 12.2 mA) (*p* < 0.05) in the pulsed US group. The *post hoc* comparisons between continuous US at 1 MHz (12.4 to 11.4 mA) and control condition (12.0 to 10.8 mA) showed no significant difference (*p* > 0.05). However, the change in perception threshold was greater after control (2.8 mA in pulsed US group and 1.6 mA in continuous US group; *p* < 0.05) than after the pulsed US (1.1 mA) or continuous US therapy (1.0 mA) (*p* < 0.05) (Table 3 and Figure 3).

### Skin blood flow over treatment area

**Figure 4:**
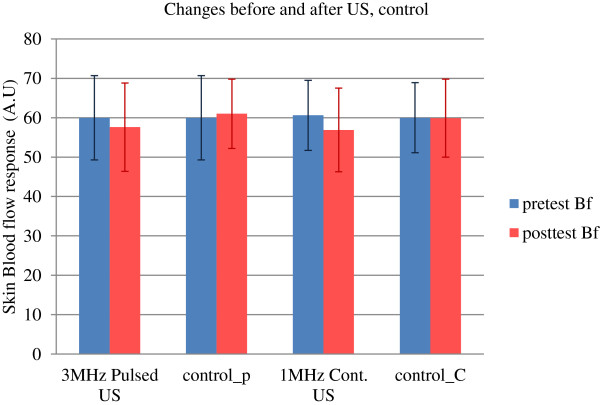
Skin blood flow response before and after US and control.

**Figure 5:**
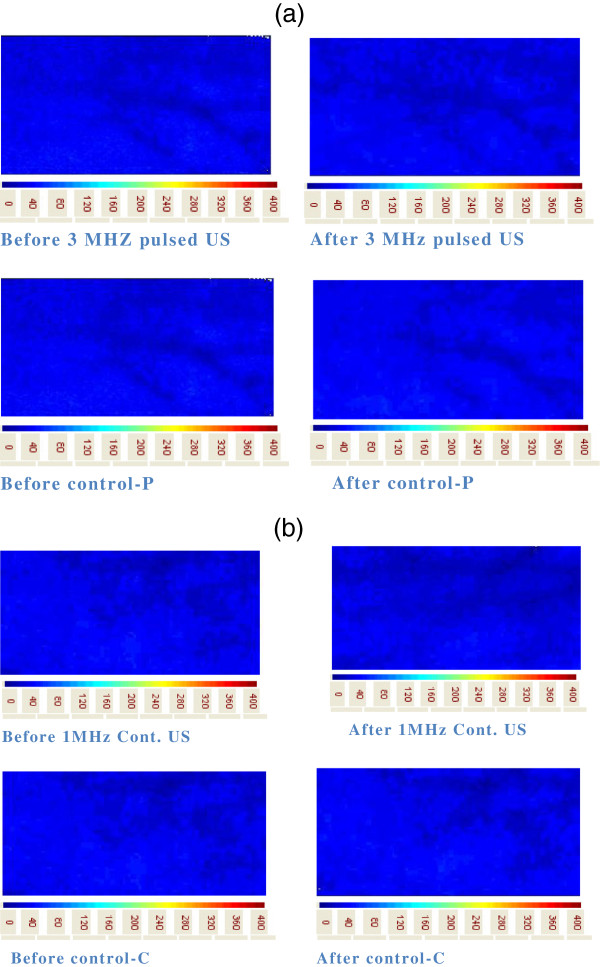
**Skin blood flow responses. (a)** Skin blood flow responses as seen on TiVi camera in the pulsed US group. **(b)** Skin blood flow responses as seen on TiVi camera in the continuous US group.

There was a significant decrease in skin blood flow over the treatment area from baseline to post-test after the ultrasound (1 or 3 MHz) and control condition (*p* < 0.05). A significant interaction was also found between time and treatments (*p* < 0.05). *Post hoc* comparisons revealed that pulsed US at 3 MHz resulted in a statistically significant decrease in superficial blood flow (60.3 to 57.6 AU) as compared to that of the control condition (60.3 to 60.1 AU) (*p* < 0.05) in the pulsed US group. Similarly, the *post hoc* comparisons between continuous US at 1 MHz showed a significant decrease in blood flow (60.6 to 56.9 AU) as compared to that in the control (60.6 to 59.9 AU) (*p* < 0.05) in the continuous US group. The change in blood flow was very small after control (1.1 AU in pulsed US group and 1 AU in continuous US group; *p* < 0.05) than after the pulsed US (2.5 AU) or continuous US therapy (3.5 AU) (*p* < 0.05) (Table 2 and Figures 3, 4, and 5).

### Skin temperature over the area of treatment

**Figure 6:**
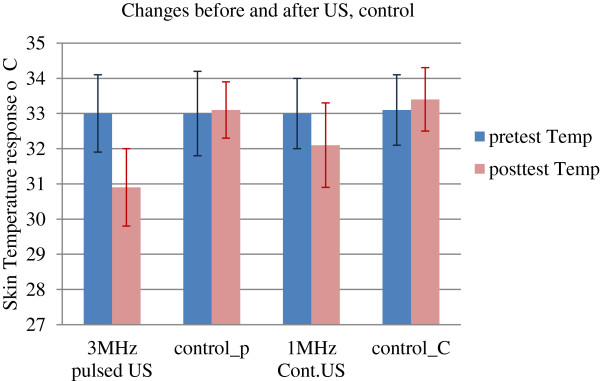
Skin temperature response before and after US and control.

A significant decrease in temperature was also observed over the treatment area from pretest to post-test with ultrasound (1 or 3 MHz) as well as control condition (*p* < 0.05). A significant interaction was also found between time and treatments (*p* < 0.05). *Post hoc* comparisons revealed that pulsed US at 3 MHz resulted in a statistically significant decrease in skin temperature (33.0°C to 30.9°C) when compared to the control condition over the treatment area (33.1°C to 33.0°C) (*p* < 0.05). Similarly, the *post hoc* comparisons between continuous US at 1 MHz showed statistically significant decrease in skin temperature (33.1°C to 32.1°C) when compared to the control condition (33.4°C to 33.1°C) (*p* < 0.05). The change in temperature was very small with control (0.05°C in pulsed US group and 0.3°C in continuous US group) than that after the pulsed US (2°C) or continuous US (1.5°C) (*p* < 0.05). However, participants reported that they felt the skin and the transducer head became slightly warmer during or after continuous US (1 MHz) but did not report this during or after pulsed US therapy (3 MHz) (Table 2 and Figure 6).

### Effect of age and gender

Analysis with age and gender as covariates revealed no significant effect of age (across the two categories; 18–34, 35–50 age groups) as well as the gender on the R-CPT scores or superficial palmar blood flow or skin temperature (*p* > 0.05) after US therapy.

## Discussion

Ultrasound therapy (3 MHz, pulsed US and 1 MHz, continuous US) demonstrated a small to moderate effect on skin blood flow (RBC concentration), skin temperature and sensory perception thresholds at 5 Hz (C fibers) over the treatment area in the distal forearms of healthy volunteers. This study resulted in a significant reduction in skin blood flow, skin temperature, and C fiber perception thresholds immediately after the 3 MHz pulsed US, 1 MHz continuous US, and control (rest). Whereas, the sensory perception thresholds at 2,000 Hz (A-beta fibers) remained unaltered by all three conditions (3 MHz, pulsed US, 1 MHz, continuous US, and rest).

Previous studies have demonstrated that US has the potential to decrease blood flow in rat models [44-46] and human calf skin [47]. In the present study, the results demonstrated that application of US (whether pulsed or continuous) significantly decreases skin blood flow immediately after therapy, thus supporting the work by Ware et al. There was a decrease in skin blood flow over time without the treatment (control/rest) as well, but the changes observed were smaller (≤1.0 AU) when compared to continuous US (3.5 AU) or pulsed US therapy (2.5 AU). Our findings are consistent with Ware et al. who reported an average decrease of 12% in dermal blood flow and elevated skin temperatures averaging 1.4°C with continuous, 3 MHz US, using different measurement and treatment procedures. They used laser Doppler flowmetry measured from calf muscles, and a higher US dosage was implemented including an intensity of 1.5 W/cm^2^ and a duration of 10 min [47]. Thus, the total energy delivered was quite different than what was used in this current investigation. In contrast, our study found decreased skin temperature along with decreased skin blood flow immediately after the US application (both pulsed and continuous US) (Figure 6). The current findings on temperature in our study are similar to the previous reports which have revealed that skin temperature decreases with US intervention [48-50]. Bickford and Duff and Paul and Lmig attributed their findings to the use of a water-cooled applicator for the data collection [48,49], while Kramer attributed the temperature changes to US transmission gel [50]. It is known that when in contact with cold materials, the skin tends to freeze at higher temperatures than when exposed only to cold air due to a reduction in the amount of supercooling [51]. This may further be explained by the thermal responses to local cooling of skin (mild), which stimulates the cold-sensitive afferents to activate sympathetic nerves to release norepinephrine, leading to a local cutaneous vasoconstriction [26,52]. Thus, the underlying mechanism of the demonstrated changes in superficial blood flow and skin temperature in this study might have been due to the US transmission gel [50] and the metal plate of the US transducer head [48,49].

Kramer demonstrated a linear relationship between subcutaneous tissue temperature and US intensity [50]. In their study, subcutaneous temperature increased linearly with increasing intensity after 5 min of sonation, using a frequency of 0.87 MHz. These authors [47] observed a significant decrease in skin temperature during the first minute of sonation at 0.0 and 0.5 W/cm^2^ demonstrating a rapid cooling effect and also during the recovery periods at 1.5, 2.0, and 2.5 W/cm^2^ after the gel was wiped off from the skin. It was not until US intensity had been increased to 2.0 W/cm^2^ that the thermal heating effect of US negated the immediate superficial thermal-cooling effect of the gel to produce significantly increased subcutaneous tissue temperature during sonation. It was suggested that, as US provided a deep heating effect, the US transmission gel on the skin surface provided a superficial cooling effect [50]. In the present study, we too observed a similar temperature response to US therapy. Skin temperatures decreased with 0.25 W/cm^2^, 3 MHz pulsed US as well as with 0.8 W/cm^2^, 1 MHz continuous US, but the largest drop in skin temperature was seen with 3 MHz pulsed US at 0.25 W/cm^2^ (2°C *p* < 0.05) and not with the continuous US at 0.8 W/cm^2^ (1.5°C, *p* < 0.05) or with control/rest (≤ −0.3°C, *p* < 0.05). Therefore, it is suggested that there will be minimal cooling in skin after the US application if the cooling of the gel was counteracted by continuous US or with use of higher intensities.

The current study demonstrated that there was no change in A-beta perception threshold in the ring finger after the application of pulsed US, continuous US, or after control/rest (Figure 2). We could not find any study that has looked at the sensory perception thresholds before and after US therapy in the distal forearms of healthy volunteers. However, there are some previous reports on median and radial nerve sensory nerve conduction velocities (sNCV) observed after US in healthy subjects using variable intensities for different durations [52-55]. Because conventional nerve conduction studies can measure the conduction velocities of large diameter nerve fibers [42,43], the responses most similar in our study would be the 2,000-Hz R-CPT scores. Andrew et al. [52] found no significant differences in the median nerve sNCV when comparing the experimental groups (at three intensities: 0.5, 1.0, and 1.5 W/cm^2^) and control group (no US) with the placebo group (0.0 W/cm^2^) after 10 min of US therapy [52]. Therefore, it could be assumed that the application of ultrasonic waves, either pulsed or continuous, has no effect on the touch and pressure sensations in the area supplied by median and ulnar nerves.

The present study also demonstrated that there was a decrease in C fiber perception threshold in the ring finger after the application of pulsed US, continuous US, or after control/rest (Figure 3). We could not find any study which looked at the sensory perception thresholds after US therapy. But we can relate these findings to the previous reports which have demonstrated that peripheral nerve conduction responses after US vary according to the subtype of nerve fibers. Several investigators have shown that ‘B’ peripheral nerve fibers are most sensitive to US, followed by ‘C’ fibers, while ‘A’ nerve fibers were the least sensitive to US therapy [56-58]. Decrease in sensory perception threshold corresponds to hypersensitivity or increased sensitivity; hence, we can presume that changes observed in this study might have been due to the increased responsiveness of C fibers to US therapy. These changes in C fiber perception threshold may be thought to help with pain modulation [59]. C fibers transmit polymodal nociceptive, slow pain, temperature sensations, and carry postganglionic sympathetic signals; hence, the decrease in sensory perception thresholds associated with pulsed US and continuous US might have been influenced by changes in skin temperature through a thermal-cooling effect of the US transmission gel or by the ultrasound itself. The temperature of the forearm skin was comparatively higher than the treatment area (2× ERA) where gel was applied; hence, we can presume that this temperature difference caused a mild cooling effect on skin and lead to the stimulation of sympathetic vasoconstriction and thus reduced blood flow and temperature as mentioned earlier. These immediate vasoconstrictor responses after mild local cooling of skin requires both intact sensory and sympathetic functions and are thought to manifest through a complex combination of sensory, autonomic, and direct effects. This can further be explained by several underlying mechanisms, involving effects on receptor translocation, transmitter secretion and vascular smooth muscle contractile function [26].

Differing construct parameters, methods of blood flow measurement, skin temperature measurement, and treatment protocols do not allow direct comparisons to be made between the research studies that have been published to date [30,47-50,52,60,61]. However, the observed changes with US therapy are deductible from the previous physiological findings, but the underlying mechanism of the demonstrated changes in skin blood flow, skin temperature, and sensory perception thresholds obtained with 5 Hz stimulation in the control are still unclear. The small changes observed in skin temperature and skin blood flow after the control or rest period (3 to 5 min) might have been due to the emotional factors, which were not under participants’ control. Whereas the changes in sensory perception threshold at 5 Hz after control/rest in both groups (3 MHz pulsed or 1 MHz continuous US group) may have been due to the ‘wind-up phenomenon’ [62], which is described as a frequency-dependent increase in the excitability of spinal cord neurons in response to the C fiber activity (repetitive stimulation) [62]. The lowering of sensory perception thresholds at 5 Hz in this study following repetitive stimulation of C fibers is consistent with the previous neurometer CPT findings of Wallace et al. and Farajidavar et al. in the control group [62,63]. The changes observed after control condition can also be explained by order effect or learning effect from the repeated testing on the neurometer. TiVi and infrared skin thermometer are objective outcome measures, and they could not have influenced the participants’ blood flow or temperature responses while at rest. But the neurometer is both subjective [40] (subjects have to respond to 5 Hz stimuli and stop the test when they begin to feel the tingling or buzzing sensation near the electrodes) and objective assessment tool [40] and this might have had an influence on the observed threshold responses. At the beginning, participants were unaware of the transcutaneous electrical stimulation from the neurometer and might have responded with higher thresholds (higher baseline values), but as the tests continued and repeated several times, they become familiar with the test and type of stimulations thus performing subsequent tests more fast and more conveniently, indicating a possible learning effect [64].

It is also known from previous reviews that therapeutic ultrasound is often used as a deep heating rehabilitation modality for hand conditions [4,6]. The present investigation only examined the effect of ultrasound from superficial tissues in the distal forearms. TiVi can reflect red blood cell concentrations from the skin at a depth of 0.4 to 0.5 mm (well into the reticular dermis), while the digital infrared thermometer and R-CPT tests record temperature and sensation superficially. Adequate washout was attained before each pre-test for all measures. However, it is not known, whether the dosage used for 3 MHz pulsed US and 1 MHz continuous US in this study actually increased or decreased the tissue blood flow and tissue temperatures at a depth of 2 cm (for 3 MHz US) and 3–5 cm (for 1 MHz US ) or had no effect at all. Bickford and Duff reported some conflicting responses like decreased subcutaneous and skin temperatures (superficial) along with increased muscle blood flow (at a depth of 1 to 3 cm) after sonating healthy tissues with 0.8 MHz, using higher intensities (ranging from 2.0–3.0 W/cm^2^) [47,48]. These authors [47] demonstrated that consistent sustained increase in blood flow and tissue temperature in the deep muscles occur only with higher intensities of US treatment (over 3.0 W/cm^2^), while the moderate intensities causes reduction in skin blood flow and skin temperature due to the effect of contact cooling with US applicator [47]. Lehmann and Delateur reported that vigorous heating requires higher total power outputs (up to 40 W) and higher US intensities (up to 4 W/cm^2^) [65]. Most joints covered with a significant amount of tissue (shoulder, hip) may be moderately heated with a total power output of 10 to 20 W (at 1 to 2 W/cm^2^ US intensity) [65]. For very mild treatments, or for small joints with minimal soft tissue cover, a total output of 1 to 10 W (at 0.1–1.0 W/cm^2^ US intensity) may be used for adequate heating [53]. Though tissue heating is regarded as beneficial, Merrick et al. cautioned that it is difficult to predict temperature increases when ultrasound is applied clinically owing to a number of unknown variables, including the distance to reflecting soft tissue-bone interfaces, variability among ultrasound machines, the thickness of each tissue layer, and the amount of circulation [12,18,66,67].

In summary, it is assumed that 3 MHz pulsed and 1 MHz continuous US at low intensities (0.25 and 0.8 W/Cm^2^ ) and short durations (5 and 3 min) in the current study might have accounted at least in part for the observed changes in skin blood flow, skin temperatures, and C fiber perception thresholds, thus indicating that US is not ineffective as a treatment modality but can have some minor effects on the superficial tissues and C fibers of sensory nerves.

Our study does not provide an indication about a therapeutic dosage. Another issue to be considered in our dosage was that we were sonating normal tissue, and the physiological effects might be more dramatic if we were dealing with injured tissue. We elected to study the physiological effects in normal tissue because of a lack of research in this area and it is important to start with a simpler construct. Further, ethics boards are often unwilling to allow research on human subjects with injuries or disease until the testing has been applied to normal individuals. This would also suggest that future studies looking at physiological effects of ultrasound on blood flow, temperature, and sensory function should move towards investigating larger dosages and patient populations (damaged tissue).

### Limitations and research recommendations

There are a number of limitations in the current study that may have affected the study findings and generalizability. Only one therapist provided the treatment and assessments, which meant that the evaluator was not blinded. However, three of these measures were not under control of the therapist. The sensory threshold was determined by the participant while the TiVi and thermometer were not controlled by either the participant or therapist, so minimal bias was expected with respect to outcome evaluation. However, the TiVi system while accurate is only able to measure superficial blood flow and the US effects in deeper tissue were not measured.

For the sensory evaluation, we used the rapid CPT assessment which has less repetition than the full CPT protocol, which involves a repeated force choice protocol. While this may have made our sensory measurements less precise, it was deemed appropriate given we were looking for transient changes and the forced choice protocol can be more time-consuming and we may have lost the opportunity to see the short-term changes. The sensory perception thresholds were recorded from the tip of ring fingers (dermatome supplied by C7 and C8), instead of the treatment area in order to follow the recommended guidelines of the manufacturer, and to avoid any unwanted motor point stimulation (muscle contraction) and thus may not reflect sensory changes in the treatment area. Furthermore, since the ring is innervated by both the median and ulnar nerves, differential effects in these nerves were not directly explored.

Real-time measurements of skin blood flow and skin temperature over the treatment area during the application of US was not possible because of the continuous movement of transducer head which was in-turn blocking the view of TiVi images. This is considered a minimal limitation since very transient effects are unlikely to have therapeutic value.

As there is no standard for US dosage, the two US doses (3 MHz pulsed and 1 MHz continuous) selected in this study were based on the empirical evidence and therapeutic principles of US therapy [20,33]. Hence, there is a need to develop US therapy dosage guidelines for different disorders.

The responses observed in this study suggest that small to moderate changes in skin blood flow, skin temperature, and sensory nerve perceptions should be expected with brief exposure to US therapy. Hence, there is a possibility that the observed changes may have some beneficial effect on the injured tissues. Knowledge about the short-term and long-term physiologic effects of US also informs our understanding of the safety and therapeutic benefit of US therapy. Thus, future research should explore the effects of US in patients with hand injuries and with comorbid health problems using different dosages commonly used in the clinical practice. Furthermore, responses from US can be compared with the responses from other physical agent modalities commonly used in hand rehabilitation to help choose the best treatment for patients.

## Conclusions

In conclusion, this study demonstrated that both 3 MHz pulsed (1:4, 0.25 W/cm^2^, 5 min) and 1 MHz continuous US (continuous, 0.8 W/cm^2^, 3 min) significantly decreased skin blood flow, skin temperatures, and C fiber perception thresholds immediately after US therapy. There was no significant change in A-beta perception thresholds, detecting touch, and pressure sensations with US therapy, indicating the negligible effect of US on these fibers. The changes observed in C fiber perception thresholds after US indicate that these fibers are more sensitive to US therapy and may have a potential role in influencing the pain signals carried by C fibers which needs further investigation. It is presumed that all these physiologic responses observed could have been due to a sympathetic vasoconstrictor effect as a result of thermal changes (thermo-cooling effect of gel and transducer head) or biochemical tissue responses to ultrasound or due to a direct effect of US on C fibers belonging to median and ulnar nerves. However, further research using different US dosages commonly used in clinical practice settings would be required to definitively establish the putative therapeutic effects and underlying physiologic mechanism(s) of action of US and how they are influenced by different parameter settings.

## Competing interests

The authors declare that they have no competing interests.

## Authors’ contributions

The concept and design, acquisition of data, and analysis and interpretation of data described in this study were carried out by SS. JM also conceived the study and provided her expertise and support. SS prepared the initial draft, which was revised by JM, TB, RG, and BF. All authors read and approved the final manuscript.
